# The prevalence of pulmonary complications after thoracic and abdominal surgery and associated risk factors in patients admitted at a government hospital in Harare, Zimbabwe-a retrospective study

**DOI:** 10.1186/s13741-017-0066-3

**Published:** 2017-08-22

**Authors:** Cathrine Tadyanemhandu, Rufaro Mukombachoto, Clement Nhunzvi, Farayi Kaseke, Vasco Chikwasha, Samson Chengetanai, Shamila Manie

**Affiliations:** 1 Department of Rehabilitation, College of Health Sciences, University Of Zimbabwe, PO Box AV 178, Avondale, Harare, Zimbabwe; 2 Department of Community Medicine, College of Health Sciences, University Of Zimbabwe, PO Box AV 178, Avondale, Harare, Zimbabwe; 3 Division of Basic Medical Sciences, Faculty of Medicine, National University of Science and Technology, PO Box AC 939, Ascot, Bulawayo, Zimbabwe; 4 Division of Physiotherapy, Department of Health and Rehabilitation Sciences, Faculty of Health Sciences, University of CapeTown, Anzio Road, Observatory, CapeTown, South Africa

**Keywords:** Abdominal surgery, Thoracic surgery, Post-operative complications, Pulmonary complications, HIV infection, Comorbidities

## Abstract

**Background:**

The burden of HIV/AIDS in Sub-Saharan Africa has presented unusual and challenging acute surgical problems across all specialties. Thoraco-abdominal surgery cuts through muscle and thereby disrupts the normal anatomy and activity of the respiratory muscles leading to reduced lung volumes and putting the patients at greater risk of developing post-operative pulmonary complications (PPCs). PPCs remain an important cause of post-operative morbidity, mortality, and impacts on the long-term outcomes of patients post hospital discharge. The objective of the study was to determine the pulmonary complications developing after abdominal and thoracic surgery and the associated risks factors.

**Methods:**

A retrospective records review of all abdominal and thoracic surgery patients admitted at a central hospital from January 2014 to October 2014 was done. Data collected included demographic data, surgical history, comorbidities and the PPCs present.

**Results:**

Out of the 92 patients whose records were reviewed, 55 (59.8%) were males and 84 (91.3%) had abdominal surgery. The mean age of the patients was 42.6 years (SD = 18.4). The common comorbidities were HIV infection noted in 14(15.2%) of the patients and hypertension in 10 (13.0%). Thirty nine (42.4%) developed PPCs and the most common complications were nosocomial pneumonia in 21 (22.8%) patients, ventilator associated pneumonia in 11 (12.0%), and atelectasis in 6 (6.5%) patients. Logistic regression showed that a history of alcohol consumption, prolonged surgery, prolonged stay in hospital or critical care unit, incision type, and comorbidities were significant risk factors for PPCs (*p* < 0.05). The mortality rate was 10.9%.

**Conclusion:**

PPCs like nosocomial and ventilator associated pneumonia were common and were associated with increased morbidity and adversely affected clinical outcomes of patients. HIV and hypertension presented significant comorbidities which the health team needed to recognize and address. Strategies to reduce the occurrence of PPCs have to be implemented through coordinated efforts by the health practitioners as a team during the entire perioperative period.

## Background

It is estimated that surgical conditions account for 11% of the total global burden of disease and 25 million disability-adjusted life years (DALYs) in Africa, the region with the highest concentration of surgical DALYs (38/1000 population) (Ozgediaz and Riviello 2008; Ärnlöv 2016). According to the World Health Organization (WHO), DALY is a measure of overall disease burden, expressed as the number of years lost due to ill-health, disability or early death. Human Immunodeficiency Virus (HIV) infection complicates surgical practice by increasing the occurrence of surgery related disease conditions, presenting challenges in the surgical management of HIV infected patients and necessitating the introduction of appropriate methods to improve surgical outcomes (Odimba 2010). Furthermore, these patients also present with other comorbidities such as hypertension, diabetes, and tuberculosis which further complicate their post-operative management (Hanekom, Coetzee and Faure 2006).

Surgical and trauma patients invariably experience a sudden and profound systemic insult that often leads to decompensation of subclinical conditions and sets the stage for new and potentially life-threatening complications (Sawyer and Leon 2010). In Nigeria, the majority of post-surgical complications were related either to surgery or anesthetic care, leading to a very high mortality rate in surgical units (Okafor 2009). Post-operative complications, involving the respiratory tract remain the most frequent cause of morbidity contributing to significant increases in patient discomfort, increased length of hospital stay (LOS), increased use of resources and overall hospital costs as well as mortality (Brooks-Brunn 1995; Arozullah et al., 2001; Hulzebos et al., 2003; Reeve 2008). A post-operative pulmonary complication (PPC) is defined as “any pulmonary abnormality occurring during the postoperative period and resulting in clinically significant disease or dysfunction, adversely affecting the clinical course of the primary condition” (Sharafkhaneh et al. 2008) and the specific complications were defined by Jammer et al., (2015).

Patients undergoing abdominal and thoracic surgery are most prone to PPCs since the incidence of PPCs is inversely related to the distance of the surgical incision from the diaphragm (Rudra and Das 2006). Besides the type of surgery, increased number, and complexity of thoraco-abdominal surgical procedures, increasing severity of illness and age of patients undergoing surgery are all directly related to the patients’ post-surgical outcomes (Hulzebos et al., 2003) and to the incidence of PPCs. It is therefore important to understand the most common PPCs in our population so that preoperative assessment can partly focus on the risk factors for the PPCs. The main aim of this study was to determine the pulmonary complications developing after abdominal and thoracic surgery and the associated risk factors. It is envisaged that knowledge of such will help put strategies in place to minimize the occurrence of the complications as well as effectively manage them thus reducing morbidity and mortality rates.

## Methods

### Study setting

A retrospective records review was carried out at Parirenyatwa Group of Hospitals, the largest referral public hospital in Zimbabwe with a bed capacity of 1800. The records were accessed through the Medical Records Department, which stores the patients’ records in both electronic and hard-copy formats. Patients referred to this hospital are usually from the low to middle socioeconomic classes.

### Participants

Participants were selected from patients aged 16 years and older who had undergone abdominal and cardiothoracic surgery between January and October 2014 inclusive. Ethical approval was granted by the Joint Research Ethics Committee for the University of Zimbabwe and Parirenyatwa Group of Hospitals (JREC: 270/14) and the Medical Research Council of Zimbabwe (MRCZ/B/738). Permission to access patients’ records from the medical records department was also sought from the Institutional Review Board.

### Sample size

The sample size required to establish the prevalence of PPCs which were anticipated to be present in 10% of the patients (Kelkar 2015), with a precision of 5% was 87, at 90% confidence level (Epi Info Version 7.1.5.0). This was based on a population of 800, the estimated number of surgical admissions over a 10-month period.

### Data collection

Qualifying patient records were identified from the admission and ward order books of the cardiothoracic and general surgery wards. Patient hospital numbers were used to access their electronic files from the Medical Records Department. The checklist used to document data during the record review comprised of the following variables which were selected based on a review of studies on risk factors for PPCs: demographic data (age, gender, marital status, smoking and alcohol consumption history), (up to one drink per day for women and up to two drinks per day for men) medical/surgical information (specific diagnosis, cause, surgery done, category of surgery, type of procedure, length of surgery, type of incision), clinical information (ICU admission, duration of period on mechanical ventilation, length of stay in ICU, length of hospital stay, complications, comorbidities, medication, chest X-rays results, FBC results, temperature, sputum analysis, number of pre and post-operative physiotherapy sessions received) and post-operative pulmonary complications present. The incision type was divided into three groups which included upper, midline (vertical incisions) and lower incision (transverse incisions). An upper incision was described as consisting of thoracic and other incisions above the umbilicus such as thoracoabdominal incision, median sternotomy, posterolateral, anterolateral, axillary, anterior, and posterior thoracotomies. Midline/vertical incisions included the midline and paramedian incisions and the Mayo-Robson extension of the paramedian incision. Lower or transverse incisions included the Kocher, Transverse, Rockey-Davis, Maylard, and Pfannenstiel incisions.

### Data analysis

Data was analyzed using Stata (Version 13). Continuous data was summarized using means and standard deviations whereas categorical data was reported as frequencies and percentages. Chi-square test and Fisher’s exact were used to test for association. Logistic regression was used to determine the risk factors for development of the PPCs in the patients. For multivariate analysis, variables were selected for inclusion into multiple logistic regression model based on *p* < 0.25 in the univariate analysis. The level of significance was set at *α* < 0.05.

## Results

### Demographics of the participants

A total of 92 patient records were accessed from Parirenyatwa Hospital Medical Records Department, 84 (91.3%) of whom had undergone abdominal surgery and 8 (8.7%) thoracic. Fifty-five (59.8%) patients were male and the overall mean age of all participants was 42.6 ± 18.4 years. Table 1 shows the demographic characteristics of the patients.

**Table 1:** Demographics and clinical information for the patients (*n* = 92)

Characteristics	PPCs present	PPCs absent
Sample size, *n* (%)	39 (42.4)	53 (57.6)
Age, mean(SD)	45.8 (17.2)	40.0 (19.1)
Gender-males, *n* (%)	26 (66.7)	29 (54.7)
Duration of surgery/min, mean(SD)	148.8 (75.0)	82.7 (35.7)
Length of CCU stay/days, mean(SD)	6.4 (7.3)	0.6 (1.3)
Hospital stay/days, mean(SD)	14 (7.6)	6.3 (7.7)
Cause of surgery, *n* (%)		
Disease	35 (89.7)	47 (88.7)
Accidents	4 (10.3)	6 (11.3)
Type of surgery, *n* (%)		
Abdominal	34 (87.2)	50 (94.3)
Cardiothoracic	5 (12.8)	3 (5.7)
Types of comorbidities, *n* (%)		
None	18 (46.1)	41 (77.4)
HIV	9 (23.1)	5 (7.5)
Hypertension	8 (20.5)	4 (7.5)
Diabetics	2 (5.1)	2 (3.7)
Previous pulmonary disease (COPD)	1 (2.6)	0
Renal disease	1 (2.6)	1 (1.9)
Post-op physiotherapy, *n* (%)		
Yes	38 (97.4)	14 (26.4)
No	1 (2.6)	39 (73.6)

### Clinical information and surgical history

The majority of surgeries done, 70 (76.1%) were emergency while 22 (23.9%) were elective. Surgery was due to disease conditions in 82 (89.1%) patients and trauma following an accident in 10 (10.9%) patients. Comorbidities were present in 33 (35.9%) patients, with HIV infection being the most common cormobidity in 14 (15.2%) followed by hypertension in 12 (13.0%) patients. The mean duration of surgery was 110.0 ± 64.0 min and patients had a mean duration stay in the critical care unit of 3.1 ± 5.6 days. The mean hospital stay for the cohort was 9.7 ± 7.3 days and the mortality rate was 10.9%. Midline incisions were the most common incisions having been done in 60 (65.2%) participants, lower incisions in 20 (21.7%) and upper incisions in 12 (13.1%).

### Postoperative pulmonary complications and associated risk factors

Out of the 92 patients, 39 (42.4%) developed PPCs. The most common PPCs in the cohort included nosocomial pneumonia in 21 (22.8%), ventilator associated pneumonia in 11 (12.0%) and atelectasis in 6 (6.5%) patients. Post-operative physiotherapy was administered to 52 (56.5%) patients, with the median number of physiotherapy sessions being 2 (IQR = 0–9.0). The majority of the patients who developed PPCs were male, 26 (66.7%) but there was no significant association between development of PPCs and gender (*X* ^2^ (1) = 1.3343, *p* = .25). History of alcohol consumption was found to be a risk factor for PPC development (*p* = 0.038) (Table 2).

**Table 2:** Demographic risk factors for postoperative pulmonary complications

Characteristic	Postoperative pulmonary complication		OR (95% CI)	*p* value
	Yes	No		
Age, mean ± sd	45.8 ± 17.2	40.2 ± 19.1	1.02 (0.99–1.04)	0.156
Gender				
*Female*	13	24	1	0.250
*Male*	26	29	1.66 (0.70–3.90)	
Smoking history				
*No*	29	45	1	0.212
*Yes*	10	8	1.94 (0.69–5.49)	
Alcohol consumption history				
*No*	23	42	1	0.038
*Yes*	16	11	2.66 (1.06–6.67)	

Patients who developed a PPC had longer duration of surgery, critical care stay as well length of hospital stay compared to those who did not and this was statistically significant for all variables (*p* < .001). The following were found to be the risk factors for developing PPCs; duration period of surgery, length of critical care stay, length of hospital stay, having a disease due to tumors and presence of comorbidities (Table 3).

**Table 3:** Univariate analysis of clinical risk factors for postoperative pulmonary complications

Characteristic	Postoperative pulmonary complication		OR (95% CI)	*p* value
	Yes	No		
Duration period of surgery (min), mean ± sd	148.8 ± 75.0	82.7 ± 35.7	1.02 (1.01–1.03)	<0.001^a^
Length of critical care stay (days), mean ± sd	6.4 ± 7.3	0.6 ± 1.3	1.73 (1.32–2.26)	<0.001^a^
Length of hospital stay (days), mean ± sd	14 ± 7.6	6.3 ± 7.7	1.36 (1.17–1.57)	<0.001^a^
Category of diagnosis				
Inflammations	17	34	1	0.634
Infective	7	10	1.33 (0.41–4.36)	
Trauma	5	3	2.00 (.36–10.97)	0.425
Tumors	10	6	3.60 (1.04–12.42)	0.043^a^
Comorbidities				
*None*	17	42	1	0.001^a^
*Present*	22	11	4.94 (1.97–12.36)	
Outcome				
*Deceased*	5	5	1	0.607
*Discharged*	34	48	0.71 (0.19–2.64)	

^a^Denotes statistically significant

Under surgery related risk factors, the type of the incision was found to be a major risk factor, especially if it was either a middle or an upper incision (Table 4).

**Table 4:** Univariate analysis of surgical related risks factors for postoperative pulmonary complications

Characteristic	Postoperative pulmonary complication		OR (95% CI)	*p* value
	Yes	No		
Cause of surgery				
*Disease*	35	47	1.12 (0.29–4.26)	0.871
*Trauma*	4	6	1	
Category of surgery				
*Emergency*	31	39	1.39 (0.52–3.74)	0.513
*Elective*	8	14	1	
Incision type				
*Lower*	2	18	1	0.005^a^
*Middle*	30	30	9 (1.92–42.23)	
*Upper*	7	5	12.6 (1.97–80.76)	0.008^a^

^a^Denotes statistically significant

Multivariate analysis was done to determine the risk factors and to control for possible confounding variables. The only factors which were statistically significant in the development of PPCs in the patients were duration of surgery and length of hospital stay (Table 5).

**Table 5:** Multiple logistic regression for risk factors for developing pulmonary complications after surgery

Characteristic	Postoperative pulmonary complication		OR (95% CI)	*p* value
	Yes	No		
Age, mean ± sd	45.8 ± 17.2	40.2 ± 19.1	0.99 (0.93–1.06)	0.826
Gender				
*Female*	13	24	1	0.857
*Male*	26	29	0.85 (0.15–4.89)	
Smoking history				
*No*	29	45	1	0.069
*Yes*	10	8	0.06 (0.00–1.24)	
Alcohol consumption history				
*No*	23	42	1	0.060
*Yes*	16	11	7.02 (0.92–53.46)	
Duration period of surgery (min), mean ± sd	148.8 ± 75.0	82.7 ± 35.7	1.03 (1.01–1.05)	0.003^a^
Length of hospital stay (days), mean ± sd	14 ± 7.6	6.3 ± 7.7	1.21 (1.03–1.42)	0.019^a^
Incision type				
*Lower*	2	18	1	0.313
*Middle*	30	30	5.06 (0.22–118.10)	
*Upper*	7	5	2.89 (0.04–194.32)	0.621
Category of diagnosis				
Inflammations	17	34	1	0.476
Infective	7	10	0.50 (0.07–3.41)	
Trauma	5	3	0.43 (0.03–6.73)	0.550
Masses	10	6	2.68 (0.28–26.03)	0.394
Comorbidities				
*None*	17	42	1	0.066
*Present*	22	11	6.01 (0.89–40.66)	

^a^Denotes statitically significant

## Discussion

Studies conducted in low-income countries showed that 10–15% of hospital admissions were due to diseases amenable to surgical treatment, of which diseases were more prevalent in the young population compared to high income countries (Hanekom, Coetzee and Faure 2006, Frankel et al., 2007, Semer, Sullivan and Meara 2010, Chalya et al., 2011, Pearse et al., 2012, Kodra, Shpata and Ohri 2016) and the same pattern was noted in the current study. The affected age group in African countries of between 15 and 44 years is the predominant economically active segment of the population. Previous reports indicated that in this age group traumatic injuries from road traffic crashes or assault were the second most common cause of morbidity and mortality after HIV infection, particularly in males (Hanekom, Coetzee and Faure 2006, Ntumba and Juma 2011). However, our study sample showed lower prevalence of HIV in both elective and traumatic surgery patients when compared to reports from other African countries (Hanekom, Coetzee and Faure 2006, Odimba 2010, Pandie et al., 2012).

The majority of patients had abdominal surgery mainly due to infection or the presence of tumors. This compared well with reports from other countries like India, Malawi, and Ethiopia where abdominal surgery was the most common surgery performed especially in the central hospitals (Bhat, Shinde and Chaudhari 2006, Lavy et al., 2007, Denu et al., 2015). The commonest causes for laparotomies in African countries have been listed as injuries, infections and neoplasms (Grimes et al., 2012, Tadyanemhandu and Manie 2015). In Zimbabwe, the prevalence of violent or injury related surgeries have been reported to be higher in the Southern part of the country, in the Matebeleland Region as compared to the Northern part (Tadyanemhandu and Manie 2015). The current study was done in the Northern part of the country, hence the small number of injury related surgeries.

Post-operative pulmonary complications were very high indicating the burden of morbidity experienced by patients undergoing thoracic and abdominal surgery in a low income country hospital. The final outcome of critically ill surgical and trauma patients depends, to a large extent, on events that take place after the original injury, surgical emergency or elective procedure (Sawyer and Leon 2010). A significant proportion of patients in our study developed various PPCs which further impacted on the duration of stay in either critical care or general hospital stay potentially worsening final outcomes.

The prevalence of PPCs in our study was found to be 42.4%. The prevalence rate for development of PPCs in our study falls in the range of 5–80% which was reported for non-thoracic surgeries by Fisher, Majumdar and McAlister (2002). The reason for this wide variation was attributed to the set-up, pre-operative and intraoperative risk factors (Rock and Rich 2003), use of different diagnostic criteria or definitions for PPCs, the type of surgery category and the type of patients recruited (Smetana 2009). The reason why our prevalence rate was relatively high might be due to the surgical specialties under consideration, since it has been reported that the prevalence of PPCs is high in abdominal and cardiothoracic surgery patients. Nosocomial pneumonia was the most common PPC identified in our study. This was similar to other studies which highlighted that pneumonia was the most common PPC as it ranked third after urinary tract infection and wound infection (Inoue et al., 2013; Denu et al. 2015). The other complications included atelectasis, respiratory failure, and pleural effusion which were also reported in other studies (Jensen and Yang 2007, Okafor 2009).

To reduce the incidence or prevalence of PPCs, there must be an understanding of patient conditions that increase the risk of developing PPCs and of effective interventions available to reduce the impact of pre-existing patient’s conditions on the subsequent development of PPCs (Rudra and Das 2006, Denu et al., 2015, Kodra, Shpata and Ohri 2016). The risk factors for development of PPCs from our study were found to be incision type, presence of comorbidities, duration of surgery, length of critical care stay, and hospital stay. These findings were also reported in other studies which looked at predictors of PPCs in patients (Rudra and Das 2006; Jensen and Yang 2007; Denu et al. 2015, Kodra, Shpata and Ohri 2016). Gender, age over 65 years and category of surgery were not found to be associated with development of PPCs in our study contrary to other studies (Jensen and Yang 2007, Denu et al., 2015, Kodra, Shpata and Ohri 2016). This may be due to a small number of patients over the age of 60 in our study as majority was below the age of 45. However, there is evidence that advanced age is an important predictor of postoperative pulmonary complications, even after adjustment for comorbid conditions (Qaseem et al. 2006). Moreover, following any type of surgery, patients are reluctant to move because of fear of damaging the incision site and the pain associated with any movement. This predisposes the patients to the complications of immobility. Development of PPCs will further result in prolongation of mechanical ventilation, CCU stay or hospital stay, and this was evident in our study during comparisons between the group which developed PPCs and the one which did not. It is assumed that this resulted in substantial economic burden on the hospital resources. Surgical patients from 28 European countries have been reported to have a median critical care stay of 1.2 days (IQR = 0.9–3.6) and a median duration of hospital stay of 3.0 days (IQR = 1.0–70), far lower than what is reported in most African countries (Hanekom et al., 2006, Chalya et al. 2011, Tadyanemhandu and Manie 2015). The mortality rate for our study was comparable to the 12.3% reported in South Africa (Hanekom et al., 2006) but lower than 32.7% reported in Tanzania. A plausible reason for the high mortality in Tanzania was the fact that the study was focused on trauma admissions only and not any other cause. Furthermore, high income countries reported very low mortality rates as exemplified by the 4% obtained in the study of 28 European nations (Pearse et al., 2012). After controlling for other variables in our cohort, the only risk factors associated with development of PPCs in our study were duration of surgery and length of hospital stay.

The most common comorbidities present in our study were HIV and hypertension. These comorbidities have been reported in South Africa (Hanekom, Coetzee and Faure 2006, Pandie et al., 2012). The greatest burden of disease in Africa comes from infectious diseases, with HIV and tuberculosis (TB) dominating the health landscape but now there is an increase in non-communicable diseases, with hypertension and diabetes dominating (Pandie et al., 2012). There is sufficient evidence to support that exercise capacity, diabetes, and HIV infection are independent risk factors for postoperative pulmonary complications (Hulzebos et al., 2003; Rudra and Das 2006). These comorbidities predispose patients to post-operative pulmonary complications as they result in patients having a poor baseline pulmonary function.

Results of our study show that none of the patients received pre-operative physiotherapy and the majority only received post-operative physiotherapy after a complication had developed. Similar findings were obtained in Australia and New Zealand where very few physiotherapists reported involvement in pre-operative physiotherapy despite the significant benefits highlighted in the literature (Reeve 2008). The short period between diagnosis and surgery has often been regarded as an inadequate period of time to sufficiently impact upon exercise capacity in order to improve the outcomes of patients post-surgery (Reeve 2008). Reductions in post-operative pulmonary complications will require development and consensus acceptance of diagnostic criteria and creation of valid standard of measurement of the complications (Romig and Dorman 2011).

### Limitations of the study and recommendations

The study design was limiting since a retrospective records review made it difficult to infer causality. The sample size was not large enough hence the need to carry out a similar study, with a bigger sample size in which data is prospectively recorded in a randomized controlled trial especially to determine if HIV status and age affect the outcomes of surgical patients. There are measures that need to be taken so that spirometry tests are done in all patients undergoing abdominal and thoracic surgery to identify the at risk population.

The other limitation was unavailability of severity scores of patients using validated scales like American Society of Anesthesiologists (ASA), Acute Physiology and Chronic Health Evaluation score (APACHE), or the Sequential Organ Failure Assessment score (SOFA). This limitation was reported in another study by Tadyanemhandu and Manie (2015), and it was recommended that the physicians and anesthetists consider using validated scales to measure the severity of the condition more objectively.

## Conclusions

Post-operative pulmonary complications were common and were associated with increased morbidity and mortality and adversely affected clinical outcomes of patients. The presence of the comorbidities which included HIV, hypertension, and diabetes further complicated the management of the patients. The risk factors associated with development of PPCs in our study were duration of surgery and length of hospital stay. Strategies to reduce the rate of the complications have to be implemented by the health practitioners as a team in order to reduce the burden of disease on the patients as well as on hospital management.

## Abbreviations

ANOVA: Analysis of variance
CCU: Critical care unit
ICU: Intensive care unit
JREC: Joint research ethics committee
MRCZ: Medical Research Council of Zimbabwe
PPC: Post-operative pulmonary complication
